# Developmental profile of Filipino children born during the SARS-COV-2 pandemic: pilot study

**DOI:** 10.3389/fpubh.2024.1426409

**Published:** 2024-10-16

**Authors:** Angel Belle C. Dy, Michelle Alexandra Edillon, Mikhaela Francesca Marietta A. Malonzo, Glenda Darlene Garcia, Alane Blythe C. Dy, Clarisse Joyce S. Espiritu, Ma. Bianca Carmela Aquino, Shannen Louise T. So, Nina Linly D. Capulong, Rizza Victoria C. Dagal, Lourdes Bernadette Sumpaico Tanchanco

**Affiliations:** School of Medicine and Public Health, Ateneo de Manila University, Pasig, Philippines

**Keywords:** early childhood, child development, COVID-19, Filipino, community

## Abstract

**Objective:**

The Philippines experienced one of the longest restriction periods during the COVID-19 pandemic. This study aimed to provide a developmental profile of 18–25 month-old children and identify factors associated with their development during their early years being born and raised during the pandemic.

**Methods:**

The study population was recruited through convenience sampling among families living in proximity to the daycare centers in Cainta, Rizal, Philippines. 116 children qualified to participate and underwent developmental screening using the Early Childhood Care and Development (ECCD) Checklist and their parents were interviewed related to demographic and social factors.

**Results:**

The mean score of the children’s Overall Development is 106.47 (*SD* = 13.43) indicating that children’s skills were within the expected range of 80–119. Girls had significantly higher mean scores compared to boys [*M*_Girl_ = 111.23, *SD*_Girl_ = 9.95 vs. *M*_Boy_ = 101.18, *SD*_Boy_ = 14.83 *t*(114) = −4.32 *p* < 0.001]. Mean scores were highest among children whose mothers completed a high school education (*M*_High School_ = 107.76, *SD*_HighSchool_ = 12.47) compared to those who have some or have completed an elementary education (*M*_SomeElem_ = 72.50, *SD*_SomeElem_ = 6.36 and *M*_Elem_ = 103.58, *SD*_Elem_ = 13.86 respectively) [*F*(2, 113) = 8.18, *p* < 0.001]. Unadjusted linear regression shows a modest increase in mean scores as the number of household members increased [*ꞵ* = 0.86, (CI: 0.02, 1.70), *t*-score (1, 113) = 2.03, *p* = 0.045].

**Conclusion:**

The developmental skills of 18–25 month-old children born and raised during the COVID-19 pandemic in an urban municipality in the Philippines are within average scores. Both hindering and protective demographic factors were identified as associated with the children’s developmental evaluation scores. It is important to acknowledge these factors and continue monitoring the children’s development and address needs among children who may need further support.

## Introduction

The COVID-19 pandemic prompted the declaration of a state of emergency in the Philippines on March 8, 2020. This brought about disruptions across different facets of society. One particularly vulnerable group affected was children. The implementation of stay-at-home directives for minors, closure of educational and recreational facilities, and shifts in parental work arrangements, significantly altered the children’s daily lives. Given that the first five years of life represent a critical phase of growth and development, numerous countries have undertaken efforts to document the pandemic’s adverse impacts on early childhood development and mental well-being (1–5). This includes observed decline in performance in cognitive, motor, and language skills, which were attributed to family-related stressors due to limitations in accessing stimulating environments and other economic and environmental adversities (1).

The pandemic induced social restrictions, shutdowns, and school closures, which introduced stressors to both parents and children. These potentially affect optimal child growth and development and compromise progress toward the Sustainable Development Goals (SDGs). Recognizing the potential long-term consequences, it becomes imperative for stakeholders to comprehensively understand the underlying factors that may lead to these outcomes (2).

Examining the literature for specific aspects of child development, the pandemic has manifested as both a detrimental and a protective factor on developmental skills, specifically expressive and receptive language, social–emotional development and motor skills (2–5). Restrictions in daily activities may lead to unhealthy levels of stress to both parents and children, which then may impede child development. For instance, increased indoor activities (e.g., screen use) may reduce motor skill development. Furthermore, adverse experiences at a young age, such as those caused by epidemics, have been previously found to pose risks on development and health at later ages. While these factors could have been present before the pandemic, the lockdown highlighted and magnified the effects of these factors on the different domains of child development (2–5).

However, increased time at home provided opportunities for more shared caregiver-child interactions. These could have resulted in more responsive caregiving, increased opportunity to partake in healthy maternal and child feeding practices, and an increased capacity to provide nurturing care (5). These factors, in turn, can serve as protective factors in the child’s language and social–emotional development, and even the health of both parent and child (5).

On the other hand, studies that focused on infants and toddlers revolved around maternal health, risks of COVID-19 transmission, and critical aspects of infant care, including breastfeeding (6–8). Such studies highlighted the importance of pre- and post-partum maternal mental health in the development of children born during the pandemic. It is suggested that maternal pandemic-related stress during pregnancy affects neurodevelopment of the infants, causing delays in the various developmental domains (9). However, the specific impact on infant and toddler neurodevelopment during the pandemic has limited documentation.

This study seeks to address a gap in the literature by presenting a developmental profile of.

Filipino children aged 18–25 months who were born and raised during the pandemic in an urban municipality in the Philippines. In addition to profiling, this study describes factors that may be associated with the development of this cohort of children.

## Methods

This cross-sectional study describes the demographic characteristics, quantifies the developmental skills of children, and identifies factors that may be associated with child development in the cohort.

### Study population

Participants were 18–25 month old children residing in Cainta, a municipality in the province of Rizal, bordering the National Capital Region in the Philippines. Historically an agricultural town, its proximity to the capital led to a rising population and urbanization in recent years. As of the 2015 census, it has become one of the most densely-populated municipalities (10).

Recruitment was done through non-probability convenience sampling from March to July 2023 through the invitation of daycare center teachers. Children who have an existing diagnosis of a developmental delay or who were receiving developmental interventions were excluded from the study.

Children were accompanied by primary caregivers who answered survey questionnaires and provided observations about the child’s behavior. A primary caregiver was identified as a parent, guardian, or person who documented themselves as someone who cared for the child regularly.

The sites were chosen based on the presence of the daycare centers catering to low to low-middle income communities and are known to use the ECCD Checklist in monitoring children as mandated by the government (11).

#### Data collection tools

##### Household information sheet

The Household Information Sheet (HIS) was a non standardized survey questionnaire verbally administered to the caregivers to collect demographic information. It collected data about household demographics, parents’ educational and occupational backgrounds, child’s health and nutrition, access to resources and caregiver mental health. Caregiver mental health was assessed using questions about loneliness, stress, and depression (12–14).

##### Early childhood care and development (ECCD) checklist

In the Philippines, it is mandated by law to develop and strengthen the early childhood care and development system for the early identification, prevention, referral and intervention for children. The ECCD Checklist was developed and validated in 2001 on 10,915 Filipino children and has since been used by local government units in public daycare centers throughout the nation. The ECCD Checklist Child’s Record 1 is the version used for children aged between 0 months to 3 years (15). The checklist contains age-appropriate developmental milestones for gross motor, fine motor, self-help, receptive language, expressive language, cognitive, and social–emotional domains. Items reflect Filipino values and practices. The ECCD manual describes gross motor as the child’s movements of the body, trunk, and leg, fine motor as movements of hands and fingers, self-help as ability to do daily activities, receptive language as understanding of words heard, expressive language as ability to express thoughts and feelings through words, cognitive as “ability to think, reason, understand concepts, and solve problems,” including precursors to early literacy and numeracy skills, and socio-emotional as “ability to respond in an age and culturally appropriate manner to social situations and interpersonal relationships.” Further information about the tool, scoring, and interpretations are available in Supplementary Appendix A and Supplementary Tables S1, S2.

Each item is either scored as 0 or 1, wherein 1 indicates that the child is observed to demonstrate the behavior. The ECCD Checklist reports scaled scores for each domain of development and a standard score for overall development. Raw scores per domain are converted into scaled scores. Range of possible raw scores for each domain are the following: 0–22 for gross motor, 0–14 for fine motor, 0–14 for self-help, 0–15 for receptive language, 0–22 for expressive language, 0–18 for cognitive, and 0–14 for social–emotional. These are then scaled to possible scores between 1 to 19. A scaled score of 7–13 indicates average development in the domain. The sum of scaled scores are converted into a standard score, to which a standard score of 80–119 indicates average overall development. Score interpretations and ranges are available in the Supplementary Tables S1, S2.

Scores for each domain are reported as scaled scores and scores for overall development are converted into and reported as standard scores.

The ECCD consists of seven subscales. The internal consistency for each subscale, as measured by Cronbach’s alpha (*α*), ranged from acceptable to good reliability. Specifically, the gross motor subscale, which included 22 items, had an alpha of 0.786. The fine motor subscale (14 items) had an alpha of 0.693, and the self-help subscale (10 items) demonstrated an alpha of 0.714. For the receptive (15 items) and expressive (22 items) subscales, alphas were 0.699 and 0.721, respectively. The cognitive subscale (18 items) had an alpha of 0.708, and the social–emotional subscale (14 items) showed an alpha of 0.698.

The internal consistency for the overall ECCD checklist, which includes a total of 115 items, was also examined and found to be within the acceptable range, with an alpha of 0.749. These values suggest that the ECCD checklist and its subscales have adequate reliability for assessing various developmental domains in early childhood (16).

### Data collection process

Data collectors underwent training from the developer of the ECCD Checklist to learn the proper administration and interpretation of the tools.

Children were tested at 13 daycare centers encompassing 10 *barangays* (smallest government unit in the Philippines) in the municipality of Cainta. Researchers followed recommended minimum public health standard protocols including wearing face masks, handwashing or hand sanitizing, and disinfecting kit materials before and between each interaction with a family. Children were allowed to enter the room and given time to warm-up to the environment and data collectors. Parents were given time to review the consent form read aloud to them or read on their own. During instances when parents were not available, grandparents accompanied the child and gave consent for participation in the study. Data collectors assisted caregivers as they completed the Household Information Sheet and observed the different skills and behaviors seen within the ECCD checklist.

### Data analysis

Descriptive analysis was done to report the developmental profile of children. All statistical analyses were performed using Stata 14.2 (StataCorp LLC, College Station, TX). To identify potential factors affecting development, scaled and standard scores were tested for associations with pre-determined household-related information found in literature individually (17–20). Association of scores with categorical variables was tested by t-test for variables with two groups (sex) and one-way analysis of variance (ANOVA) for variables with three or more groups (educational levels of parents). Association of scores with continuous variables (child’s age, age of parents, number of siblings and household members, hours of physical activity and sleep) was tested by univariate linear regression analyses. A *p*-value of less than 0.05 was considered significant.

## Results

### Demographic information

The final study sample consisted of 116 children (*n* = 55 girls, *n* = 61 boys) who met the inclusion criteria. On average, mothers and fathers were aged 29–32 years. Children had approximately 1–2 siblings, and most families lived with extended family members. Most parents completed high school or a higher level of education, and more than half of the families are classified low-income. Table 1 shows the demographic and household information.

**Table 1:** Demographic and household information of study participants.

Household information	*N*	Mean	SD	Min	Max
Mother’s age (in years)	116	29.00	5.49	15	43
Father’s age (in years)	115	31.66	7.05	19	58
Child’s age (in months)	116	20.91	1.86	18	25
Number of siblings	116	1.59	1.38	0	8
Number of household members	115	6.25	2.93	3	20
Amount of physical activity (hours in a day)	116	6.37	3.21	0.5	15
Amount of sleep (total hours in a day)	116	12.50	2.00	6	17

Mother’s education	*n*	Mean	SD	Percent
Some elementary education or less	2	72.50	6.36	1.72%
Elementary school graduate	19	103.58	13.86	16.38%
High school graduate or higher	95	107.76	12.47	81.90%

Father’s education	*n*	Mean	SD	Percent	
Some elementary education or less	3	23.46	93.67	2.61%	
Elementary school graduate	21	13.18	104.29	18.26%	
High school graduate	68	12.49	106.10	59.13%	
Vocational school	9	14.90	106.67	7.83%	
College graduate or higher	14	14.08	113.57	12.17%	

Socioeconomic class*	*n*	Mean	SD	Percent
Poor (<Php 11,000)	27	107.97	12.65	23.28%
Low Income (Php 11,000 to Php 22,000)	64	105.39	13.56	55.17%
Lower Middle Class (Php 22,001 to Php 44,000)	18	108.78	12.48	15.52%
Middle Class (Php 44,001 to Php 76,000)	6	101.50	18.43	5.17%
Upper Middle Income (Php 76,001 to Php 131,000)	1	123.00	0.00	0.86%

*In terms of monthly income, based on classifications by the Philippine Institute for Development Studies.

#### Early childhood development per domain

The mean scaled scores of gross motor, fine motor, self-help, receptive language, expressive language, cognitive and social emotional were within average range of development. Self help skills scored the highest mean score at 12.06 (SD = 2.39), indicating relatively stronger performance in this area. Expressive language skills had the lowest mean score at 9.37 (SD = 2.63), suggesting comparatively weaker performance in this domain. Social–emotional skills also showed relatively lower performance with a mean score at 9.41 (SD = 3.17). Detailed scores are provided in Table 2.

**Table 2:** Mean scores (scaled scores) and standard deviation per domain of the ECCD Checklist.

ECCD scores	*N*	Mean	SD	Min	Max
**Scores per domain (scaled)**					
Gross motor	116	11.28	2.31	3	15
Fine motor	116	10.68	2.10	6	15
Self-help	116	12.06	2.39	3	15
Receptive language	116	11.68	2.57	3	15
Expressive language	116	9.37	2.63	3	17
Cognitive	116	11.78	1.96	5	15
Social–emotional	116	9.41	3.17	2	13
**Overall development**					
Sum of scaled scores	116	76.28	10.75	45	95
Standard scores	116	106.47	13.43	68	129

#### Overall early childhood development

The mean standard score for the children’s Overall Development is 106.47 (SD = 13.43). Based on the results, 35 children (30.2%) performed below average in at least one domain and need to be re-screened after 3–6 months. Supplementary Tables S1, S2 show the frequency of children’s scores for overall development and individual domains.

Statistical analyses by *t*-test or one-way ANOVA shows that there is a significant difference between the overall development scores of girls and boys [*M*_Girl_ = 111.23, *SD*_Girl_ = 9.95 vs. *M*_Boy_ = 101.18, *SD*_Boy_ = 14.83, *t*(114) = −4.32, *p* < 0.001],and among children based on maternal education (some elementary education *M*_SomeElem_ = 72.50, *SD*_SomeElem_ = 6.36), completed elementary education or some high school *M*_Elem_ = 103.58, *SD*_Elem_ = 13.86, completed high school education or higher [*M*_High School_ = 107.76, *SD*_HighSchool_ = 12.47, *F*(2, 113) = 8.18, *p* < 0.001]. The number of household members at home [*ꞵ* = 0.86 (CI: 0.02, 1.70), *t*-score(1, 113) = 2.03, *p* = 0.045] is associated with the overall development of the children by univariate linear regression (Supplementary Table S3).

#### Comparison among groups by sex and parent education

##### Sex

Of the seven subscales in the checklist, girls scored significantly higher compared to boys in all but gross motor skills, where differences were unremarkable (Supplementary Table S4).

##### Parent education

When comparing scaled scores of children based on mother’s level of education, children whose mothers attained at least a high school education or higher had the highest mean scaled score in the self-help [*M*_SomeElem_ = 8.00 *SD*_SomeElem_ = 4.24, *M*_Elem_ = 11.89 *SD*_Elem_ = 1.97, *M*_High School_ = 12.18 *SD*_High School_ = 2.39, *F*(2, 113) = 3.15, *p* = 0.046], receptive language [*M*_SomeElem_ = 5.00 *SD*_SomeElem_ = 1.41, *M*_Elem_ = 11.21 *SD*_Elem_ = 2.53, *M*_High School_ = 11.92 *SD*_High School_ = 2.40, *F*(2, 113) = 8.47, *p* < 0.001], cognitive [*M*_SomeElem_ = 7.50 *SD*_SomeElem_ = 3.54, *M*_Elem_ = 10.68 *SD*_Elem_ = 2.71, *M*_High School_ = 12.09 *SD*_High School_ = 1.56, *F*(2, 113) = 10.49, *p* < 0.001], and social–emotional domains [*M*_SomeElem_ = 3.50 *SD*_SomeElem_ = 2.12, *M*_Elem_ = 8.74 *SD*_Elem_ = 3.54, *M*_High School_ = 9.67 *SD*_High School_ = 2.99, *F*(2, 113) = 4.50, *p* = 0.01]. When comparing among groups based on father’s level of education, performance only differed in the cognitive domain [*M*_SomeElem_ = 11.00 *SD*_SomeElem_ = 1.73, *M*_Elem_ = 10.57 *SD*_Elem_ = 2.68, *M*_High School_ = 11.97 *SD*_High School_ = 1.70, *M*_Vocatio*n*_ = 12.11 *SD*_Vocatio*n*_ = 1.69, *M*_College_ = 12.50 *SD*_College_ = 1.40, *F*(4, 110) = 3.03, *p* = 0.02], where children whose fathers attained higher levels of education had higher mean scaled scores in this domain (See Supplementary Table S4 for further details).

#### Association with household-related factors

#### Age of parents

Statistical analyses by univariate linear regression show that although both mother’s and father’s age were not significantly associated with the standard score for overall development [*ꞵ* = −0.13, *t*-score(1, 114) = −0.55, *p* = 0.58 and *ꞵ* = −0.18, *t*-score(1, 113) = −0.99, *p* = 0.32, respectively], increasing ages of both parents were associated with decreased fine motor development [*ꞵ* = −0.10, *t*-score(1, 114) = −2.78, *p* = 0.01, and *ꞵ* = −0.06, *t*-score(1, 113) = −2.17, *p* = 0.03, respectively], by univariate linear regression. Increasing father’s age was also associated with decreased expressive language scores [*ꞵ* = −0.07, *t*-score(1, 113) = −2.16, *p* = 0.03].

#### Number of household members

An increase in the number of household members was associated with increased self-help skills [*ꞵ* = 0.16, *t*-score(1, 113) = 2.15, *p* = 0.03].

#### Physical activity

Increased hours of physical activity in a day was associated with increased social–emotional skills [*ꞵ* = 0.19, *t*-score(1, 114) = 2.14 *p* = 0.04].

Gross motor skills and cognitive skills were not associated with any of the above household-related factors. Other household-related factors tested that did not impact any domain scores were number of siblings and hours of sleep. Further details for the analysis per domain are included in Supplementary Table S4.

## Discussion

This study evaluated the developmental profile of Filipino children in an urban municipality during the pandemic and identified factors that are potentially associated with childhood development of this population. The study sample displayed average skill levels across overall and specific domains, which include gross motor, fine motor, self-help, receptive language, expressive language, cognitive, social–emotional.

The mean score of the children’s Overall Development is 106.47 (SD = 13.43) indicating that children’s skills were within the expected range of 80–119. The standard deviation of 13.43 indicates a moderate level of variability in the scores, with approximately 68% of the children’s scores falling within the range of 93.04 to 119.90, based on one standard deviation above and below the mean. Our overall findings indicate that girls had significantly higher mean standard scores compared to boys [*M*_Girl_ = 111.23, *SD*_Girl_ = 9.95 vs. *M*_Boy_ = 101.18, *SD*_Boy_ = 14.83, *t*(114) = −4.32, *p* < 0.001]. Mean scores were highest among children whose mothers completed a high school education (M_High School_ = 107.76, *SD*_HighSchool_ = 12.47) compared to those who have some or have completed an elementary education (M_SomeElem_ = 72.50, *SD*_SomeElem_ = 6.36 and M_Elem_ = 103.58, *SD*_Elem_ = 13.86, respectively) (*F*(2, 113) = 8.18, *p* < 0.001). Unadjusted linear regression shows a 0.86 increase in mean scores for each unit increase in number of household members [CI: 0.02, 1.70, *t*-score(1, 113) = 2.03, *p* = 0.045].

Based on results, the majority of children scored within average. The range of scores was wide, with children scoring below and above the average range in various domains of development. This is in contrast to the hypothesis that lower developmental scores, particularly with language skills, and increases in risk of developmental delays among young children who were born during the COVID-19 pandemic would be elicited (8, 19).

A limitation of the study is that children already diagnosed with a developmental delay or receiving developmental interventions were excluded from the study as they were not the usual enrollees in the day care classes. This could be a potential cause of bias in the sample. However, 30.2% of the children still scored below average in at least one domain. While the tool does not diagnose developmental delays, it provides recommendations for closer monitoring of development. This highlights the importance of community-based developmental surveillance and screening to support those in need (20, 21).

### Group differences

#### Sex of the child

Observations with regards to sex differences is in line with existing research, showing that girls often performed better in scales assessing child development (22, 23). This trend may be linked to increased parental interactions, as mothers often engage more in verbal communication and supportive activities with daughters compared to sons (22). The pandemic’s confinement to the home environment may have amplified these dynamics, enhancing the language and cognitive development of girls children during the critical period of development (9). Performance may also be due to biological differences. Findings from a systematic review show that the differences between sexes in early childhood language development are inconsistent across studies, where there may be differences in brain maturation patterns. Although biological differences in brain maturation might contribute to performance variations, both biological and environmental factors play a role in these observed differences (24).

#### Mother’s education

Children in the sample, who are not yet enrolled in formal schooling or daycare, rely heavily on their parents and caregivers to model and facilitate learning experiences. Mothers with higher levels of education are often better equipped to provide enriching environments, materials, and interactions at home (25). This aligns with research by UNICEF conducted in 2017, which highlights the essential role of parents and guardians in delivering nurturing, responsive, and stimulating interactions crucial for early childhood learning (26).

### Factors associated with development

#### Parental age

Previous studies explored how parental age may be related to child development. Considering the age ranges of mothers in this sample, it is possible that there were biological effects concerning the youngest and oldest mothers. Prior research has found that there were more risk factors during pregnancy, for maternal health, and fetal development among extreme maternal ages (i.e., younger than 17 and older than 40) (27). Another consideration is that as mothers delay giving birth to their first child, this may provide more time for their own schooling; therefore, a higher level of education (28). This could also apply to resources, wherein older parents are generally more able to provide for their family, both for mothers and fathers, regardless of socioeconomic status and family structure (29).

#### Association of household composition on child development

Larger households may offer enhanced support for childcare, providing both practical assistance and emotional support to primary caregivers, such as mothers (30, 31). In the context of the pandemic, this support may have been crucial. The Filipino collective culture, which values involvement of extended family, likely created a more supportive environment for child development during lockdowns (32). The presence of extended families offered additional cognitive stimulation and supervision, contributing to better developmental outcomes during the pandemic period (22, 26). Additional members in the household, whether extended family or the presence of siblings, may also mean increased social interactions to develop social–emotional skills, more models for learning behaviors (e.g., self-help skills such as how to use utensils) and more time and playmates for physical activities.

## Limitations

This study has limitations. First, the small sample size and confinement to a specific municipality is not generalizable to the Philippine population. The small sample size could impact the representativeness of the results, making it challenging to draw broad conclusions about the broader population. Extensive literature review for comparison data on child development utilizing the ECCD checklist on the same population was unavailable, therefore the data also lacks a comparison of child development prior to the pandemic. The authors note that the administration of the tools was done by multiple interviewers, which may introduce bias during data collection. However, given the limited sample size this was difficult to check for statistical significance.

In addition, the study excluded children with diagnosed developmental delays. This criteria may have an impact on the findings that most children performed within average expected performance. Given the context of the pandemic, this study does not look into whether mothers were affected by the illness themselves during pregnancy.

## Conclusion

This was a pilot study that described the developmental profile of Filipino children aged 18–25 months old who were born and have been growing during the pandemic in the urban municipality of Cainta, Rizal. Their developmental skills are within average scores. The study also explored group differences and factors that were associated with the development of young children in this particular community. Significant differences were observed between sexes and levels of maternal education; parental age and the number of individuals in the household had associations with the development of skills in certain domains and overall development. The COVID-19 pandemic serves as one of several environmental events that may have negative consequences on development, but it also introduced protective factors such as maternal educational attainment and continued psychosocial support in the home that supported healthy development. It is important to acknowledge these factors and continue to monitor the children’s development and target special groups who may need further support.

## Data Availability

The raw data supporting the conclusions of this article will be made available by the authors, without undue reservation.
