# Supplementary material for: Developmental profile of Filipino children born during the SARS-COV-2 pandemic: pilot study
Source: Front Public Health. 2024 Oct 16;12:1426409. doi: 10.3389/fpubh.2024.1426409 (PMC11521805; doi:10.3389/fpubh.2024.1426409)
Supplement: Supplementary file 1 [file Data_Sheet_1.PDF]

Appendix A

Further Information on the Early Childhood Care and Development (ECCD) Checklist

The study utilized the locally validated Early Childhood Care and Development (ECCD) checklist for Filipino children to assess the developmental skills of children 0 to 5 years old. This was developed in 2001 on a sample of 10,915 children in multiple regions nationwide.

The ECCD Checklist is an accessible, open-sourced, paper-based form that can be downloaded from the official website of the Early Childhood Care and Development Council (ECCDC). The ECCD Checklist Child's Record 1 is the version used for children aged between 0 months to 3 years.

The checklist contains age-appropriate developmental milestones for gross motor, fine motor, self-help, receptive language, expressive language, cognitive, and social-emotional domains. Items were created to reflect Filipino values and practices. The ECCD manual describes gross motor as the child's movements of the body, trunk, and leg (e.g. walking, running, and using stairs). Fine motor includes movements of hands and fingers (e.g.grasping objects and drawing). Self-help considers the capacity to do daily activities (e.g.feeding themselves and handling utensils). Receptive language is the understanding of words heard (e.g.understanding instructions), while expressive language is the ability to express thoughts and feelings through words (e.g.communication of needs and wants). It defines cognition as "ability to think, reason, understand concepts, and solve problems", including precursors to early literacy and numeracy skills, related to tasks like pretend play and object permanence. Lastly, social-emotional as "ability to respond in an age and culturally appropriate manner to social situations and interpersonal relationships" and can be seen through activities such as independent play and interacting with others.

Scoring

Each item is either scored as 0 or 1, wherein 1 indicates that the child is observed to demonstrate the behavior. The ECCD Checklist reports scaled scores for each domain of development and a standard score for overall development. Ranges of possible raw scores for each domain are the following: 0-22 for gross motor, 0-14 for fine motor, 0-14 for self-help, 0-15 for receptive language, 0-22 for expressive language, 0-18 for cognitive, and 0-14 for social-emotional. These are then scaled to possible scores between 1-19. A scaled score of 7-13 indicated average development in the domain.

The sum of scaled scores are converted into a standard score, to which a standard score of 80-119 indicates average overall development. Score interpretations and ranges are available in the supplement tables. (Supplement Table 1 and 2)

Interpretation

The ECCD is not a diagnostic tool but was designed to be used by caregivers, daycare teachers, local health unit workers, and volunteers who provide services to children. As such, interpretations either give recommendations (e.g. “Development in the domain must be monitored after 3/6 months”) or only suggest that a child may have advanced development.
