# Supplementary material for: Developmental profile of Filipino children born during the SARS-COV-2 pandemic: pilot study
Source: Front Public Health. 2024 Oct 16;12:1426409. doi: 10.3389/fpubh.2024.1426409 (PMC11521805; doi:10.3389/fpubh.2024.1426409)
Supplement: Supplementary file 2 [file Data_Sheet_2.PDF]

Supplement Table 1: Scaled scores **per domain** on the ECCD Checklist

Scaled Score		Interpretation
1 - 3	Below Average	Development in the domain must be monitored after 3 months
4 - 6		Development in the domain must be monitored after 6 months
7 - 13	Average	Average development
14 - 16	Above Average	Suggests slightly advanced development in the domain
17 - 19		Suggests highly advanced development in the domain

**Gross Motor Domain (Scaled Score)**	**n**	**Percentage**
Development in the domain must be monitored after 3 months	1	.86%
Development in the domain must be monitored after 6 months	1	.86%
Average development	95	81.90%
Suggests slightly advanced development in the domain	19	16.38%
**Fine Motor Domain (Scaled Score)**		
Development in the domain must be monitored after 6 months	10	8.62%
Average development	105	90.52%
Suggests slightly advanced development in the domain	1	.86%
**Self-Help Domain (Scaled Score)**	**n**	**Percentage**
Development in the domain must be monitored after 3 months	1	.86%
Development in the domain must be monitored after 6 months	4	3.45%
Average development	55	47.41%
Suggests slightly advanced development in the domain	56	48.28%
**Receptive Language Domain (Scaled Score)**	**n**	**Percentage**
Development in the domain must be monitored after 3 months	1	.86%
Development in the domain must be monitored after 6 months	6	5.17%
Average development	87	75.00%
Suggests slightly advanced development in the domain	22	18.97%

**Expressive Language Domain (Scaled Score)**	**n**	**Percentage**
Development in the domain must be monitored after 3 months	1	.86%
Development in the domain must be monitored after 6 months	16	13.79%
Average development	94	81.03%
Suggests slightly advanced development in the domain	3	2.59%
Suggests highly advanced development in the domain	2	1.72%
**Cognitive Domain (Scaled Score)**	**n**	**Percentage**
Development in the domain must be monitored after 6 months	3	2.59%
Average development	95	81.90%
Suggests slightly advanced development in the domain	18	15.52%
**Social Emotional Domain (Scaled Score)**	**n**	**Percentage**
Development in the domain must be monitored after 3 months	9	7.76%
Development in the domain must be monitored after 6 months	9	7.76%
Average development	97	83.62%
Suggests slightly advanced development in the domain	1	0.86%

Supplement Table 2: Standard Scores for **Overall Development** on the ECCD Checklist

<table>
<tr><th>Standard Score</th><th></th><th>Interpretation</th></tr>
<tr><td>69 and below</td><td rowspan="2">Below average</td><td>Overall development must be monitored after 3 months</td></tr>
<tr><td>70 - 79</td><td>Overall development must be monitored after 6 months</td></tr>
<tr><td>80 - 119</td><td>Average</td><td>Average overall development</td></tr>
<tr><td>120 - 129</td><td rowspan="2">Above Average</td><td>Slightly advanced overall development</td></tr>
<tr><td>130 and above</td><td>Highly advanced overall development</td></tr>
</table>

Overall Scores (Standard Score)	n	Percentage
Overall development must be monitored after 3 months	1	0.86%
Overall development must be monitored after 6 months	6	5.17%
Average overall development	88	75.86%
Suggests slightly advanced overall development	21	18.10%

Supplement Table 3. ANOVA (categorical variables) and univariate analysis of **demographic variables** (continuous variables) **to Overall Development** using Standard Scores

Categorical Variables	n	Mean of standard scores	Std Dev	*p* -value
**Sex**				
Male	55	101.18	14.83	< .001
Female	61	111.23	9.95	
**Mother's Education**				
Some elementary education or less	2	72.50	6.36	< .001
Elementary school graduate	19	103.58	13.86	
High school graduate or higher	95	107.76	12.47	
**Father's Education**				
Some elementary education or less	3	93.67	23.46	.12
Elementary school graduate	21	104.29	13.18	
High school graduate	68	106.10	12.49	
Vocational school	9	106.67	14.90	
College graduate or higher	14	113.57	14.08	
**Continuous Variables**	**n**	**Coeff (□)**	**SE**	***p* -value**
**Mother's Age**	116	-.13	.23	.58
**Father's Age**	115	-.18	.18	.32
**Child's Age**	116	1.49	.66	.03

**Number of Siblings**	116	-.19	.91	.84
**Number of Household Members**	115	.86	.42	.045
**Hours of Physical Activity**	116	.24	.39	.54
**Hours of Sleep**	116	.13	.63	.84

Supplement Table 4. ANOVA (categorical variables) and univariate analysis of **demographic variables** (continuous variables) **per domain** using Scaled Scores

**GROSS MOTOR**			
	**Mean (SD)**	**Statistic**	***p*-value**
**Sex**			
Male	11.20 (2.34)	t = -.37, dF = 113	.71
Female	11.36 (2.30)		
**Mother's Education**			
Some elementary education or less	9.00 (0)	F = 1.23, df = 2, 113	.30
Elementary school graduate	11.00 (2.11)		
High school graduate or higher	11.39 (2.35)		
**Father's Education**			
Some elementary education or less	11.00 (2.00)	F = 1.97, df = 4, 110	.10
Elementary school graduate	11.29 (2.22)		
High school graduate	10.93 (2.24)		
Vocational school	11.89 (2.26)		
College graduate or higher	12.71 (2.58)		
	□ (CI)	**Statistic**	***p*-value**
**Mother's Age**	.03 (-.05, .11)	t-score = .78, df = 1, 114	.44
**Father's Age**	.02 (-.04, .08)	t-score = .58, df = 1, 113	.56
**Child's Age (in months)**	.19 (-.03, .42)	t-score = 1.69, df = 1, 114	.09
**Number of Siblings**	-.21 (-.51, .10)	t-score = -1.33, df = 1, 114	.19

**Number of Household Members**	.02 (-.13, .16)	t-score = .22, df = 1, 113	.83
**Hours of physical activity (in a day)**	.07 (-.06, 2.03)	t-score = 1.05, df = 1, 114	.29
**Hours of sleep (in a day)**	-.05 (-.26, .17)	t-score = -.43, df = 1, 114	.67
**FINE MOTOR**			
	**Mean (SD)**	**Statistic**	***p*-value**
**Sex**			
Male	10.24 (2.32)	t = -2.20, df = 114	.03
Female	11.08 (1.82)		
**Mother's Education**			
Some elementary education or less	9.50 (4.95)	F = .34, df = 2, 113	.72
Elementary school graduate	10.79 (2.25)		
High school graduate or higher	10.68 (2.04)		
**Father's Education**			
Some elementary education or less	9.33 (2.89)	F = 2.22, df = 4, 110	.07
Elementary school graduate	11.00 (1.73)		
High school graduate	10.41 (2.17)		
Vocational school	10.33 (2.60)		
College graduate or higher	12.00 (1.41)		
	[illegible] (CI)	**Statistic**	***p*-value**
**Mother's Age**	-.10 (-.17, -.03)	t-score = -2.78, df = 1, 114	.01
**Father's Age**	-.06 (-.11, -.01)	t-score = -2.17, df = 1, 113	.03
**Child's Age (in months)**	.08 (-.13, .29)	t-score = .75, df = 1, 114	.46
**Number of Siblings**	-.02 (-.30, .26)	t-score = -.14, df = 1, 114	.89
**Number of Household Members**	.12 (-.01, .25)	t-score = 1.79, df = 1, 113	.08

**Hours of physical activity (in a day)**	-.06 (-.18, .06)	t-score = -1.00, df = 1, 114	.32
**Hours of sleep (in a day)**	.03 (-.17, .23)	t-score = .31, df = 1, 114	.76
**SELF HELP**			
	**Mean (SD)**	**Statistic**	***p*-value**
**Sex**			
Male	11.56 (2.42)	t = -2.16, df = 114	.03
Female	12.51 (2.30)		
**Mother's Education**			
Some elementary education or less	8.00 (4.24)	F = 3.15, df = 2, 113	.05
Elementary school graduate	11.89 (1.97)		
High school graduate or higher	12.18 (2.39)		
**Father's Education**			
Some elementary education or less	9.00 (3.46)	F = 2.31, df = 4, 110	.06
Elementary school graduate	12.29 (2.03)		
High school graduate	11.82 (2.42)		
Vocational school	12.44 (3.13)		
College graduate or higher	13.14 (1.41)		
	□ (CI)	**Statistic**	***p*-value**
**Mother's Age**	-.03 (-.11, .05)	t-score = -0.73, df = 1, 114	.47
**Father's Age**	-.04 (-.10, .03)	t-score = -1.11, df = 1, 113	.27
**Child's Age (in months)**	.05 (-.19, .29)	t-score = .39, df = 1, 114	.70
**Number of Siblings**	.05 (-.27, .37)	t-score = .33, df = 1, 114	.74
**Number of Household Members**	.16 (.01, .31)	t-score = 2.15, df = 1, 113	.03
**Hours of physical activity (in a day)**	.03 (-.11, .16)	t-score = .37, df = 1, 114	.71

**Hours of sleep (in a day)**	-.14 (-.36, .08)	t-score = -1.24, df = 1, 114	.22
**RECEPTIVE LANGUAGE**			
	**Mean (SD)**	**Statistic**	***p*-value**
**Sex**			
Male	10.87 (2.91)	t = -3.36, df = 114	.001
Female	12.41 (1.97)		
**Mother's Education**			
Some elementary education or less	5.00 (1.41)	F = 8.47, df = 2, 113	< .001
Elementary school graduate	11.21 (2.53)		
High school graduate or higher	11.92 (2.40)		
**Father's Education**			
Some elementary education or less	10.67 (4.51)	F = 1.47, df = 4, 110	.21
Elementary school graduate	10.86 (2.73)		
High school graduate	11.81 (2.39)		
Vocational school	11.11 (2.93)		
College graduate or higher	12.79 (2.42)		
	□ (CI)	**Statistic**	***p*-value**
**Mother's Age**	.06 (-.03, .14)	t-score = 1.30, df = 1, 114	.20
**Father's Age**	.02 (-.05, .09)	t-score = .63, df = 1, 113	.53
**Child's Age (in months)**	.29 (.04, .54)	t-score = 2.28, df = 1, 114	.03
**Number of Siblings**	-.03 (-.38, .31)	t-score = -.19, df = 1, 114	.85
**Number of Household Members**	.11 (-.06, .27)	t-score = 1.28, df = 1, 113	.20
**Hours of physical activity (in a day)**	.07 (-.08, .21)	t-score = .89, df = 1, 114	.38
**Hours of sleep (in a day)**	.11 (-.13, .35)	t-score = .91, df = 1, 114	.37

EXPRESSIVE LANGUAGE			
	**Mean (SD)**	**Statistic**	***p*-value**
**Sex**			
Male	8.84 (2.63)	t = -2.11, df = 114	.04
Female	9.85 (2.56)		
**Mother's Education**			
Some elementary education or less	6.00 (1.41)	F = 1.74, df = 2, 113	.18
Elementary school graduate	9.26 (2.16)		
High school graduate or higher	9.46 (2.70)		
**Father's Education**			
Some elementary education or less	9.33 (2.52)	F = .70, df = 4, 110	.60
Elementary school graduate	9.38 (3.32)		
High school graduate	9.26 (2.56)		
Vocational school	8.78 (2.11)		
College graduate or higher	10.43 (2.21)		
	□ (CI)	**Statistic**	***p*-value**
**Mother's Age**	-.06 (-.14, .03)	t-score = -1.24, df = 1, 114	.22
**Father's Age**	-.07 (-.14, -.01)	t-score = -2.16, df = 1, 113	.03
**Child's Age (in months)**	.33 (.08, .59)	t-score = 2.58, df = 1, 114	.01
**Number of Siblings**	-.28 (-.63, .07)	t-score = -1.60, df = 1, 114	.11
**Number of Household Members**	.09 (-.07, .26)	t-score = 1.09, df = 1, 113	.28
**Hours of physical activity (in a day)**	.003 (-.15, .15)	t-score = .03, df = 1, 114	.97
**Hours of sleep (in a day)**	.10 (-.14, .35)	t-score = .84, df = 1, 114	.40
**COGNITIVE**			
	**Mean (SD)**	**Statistic**	***p*-value**

**Sex**			
Male	11.22 (2.22)	t = -3.07, df = 114	.003
Female	12.30 (1.53)		
**Mother's Education**			
Some elementary education or less	7.50 (3.54)	F = 10.49, df = 2, 113	< .001
Elementary school graduate	10.68 (2.71)		
High school graduate or higher	12.09 (1.55)		
**Father's Education**			
Some elementary education or less	11.00 (1.73)	F = 3.03, df = 4, 110	.02
Elementary school graduate	10.57 (2.68)		
High school graduate	11.97 (1.70)		
Vocational school	12.11 (1.69)		
College graduate or higher	12.50 (1.40)		
	□ (CI)	**Statistic**	***p*-value**
**Mother's Age**	.01 (-.06, .07)	t-score = .21, df = 1, 114	.84
**Father's Age**	.004 (-.05, .06)	t-score .14, df = 1, 113	.89
**Child's Age (in months)**	-.02 (-.21, .18)	t-score = -.19, df = 1, 114	.85
**Number of Siblings**	.09 (-.17, .36)	t-score = .71, df = 1, 114	.48
**Number of Household Members**	.05 (-.07, .18)	t-score = .86, df = 1, 113	.39
**Hours of physical activity (in a day)**	-.02 (-.13, .10)	t-score = -.31, df = 1, 114	.76
**Hours of sleep (in a day)**	.03 (-.15, .21)	t-score = .35, df = 1, 114	.72
**SOCIAL-EMOTIONAL**			
	**Mean (SD)**	**Statistic**	***p*-value**
**Sex**			
Male	8.24 (3.50)	t = -4.05, df = 114	< .001

Female	10.48 (2.41)		
**Mother's Education**			
Some elementary education or less	3.50 (2.12)	F = 4.50, df = 2, 113	.01
Elementary school graduate	8.74 (3.54)		
High school graduate or higher	9.67 (2.99)		
**Father's Education**			
Some elementary education or less	5.33 (5.77)	F = 1.74, df = 4, 110	.15
Elementary school graduate	9.00 (3.13)		
High school graduate	9.46 (3.10)		
Vocational school	9.56 (3.57)		
College graduate or higher	10.43 (2.31)		
	□ (CI)	**Statistic**	***p*-value**
**Mother's Age**	-.05 (-.15, .06)	t-score = -.85, df = 1, 114	.40
**Father's Age**	-.05 (-.13, .04)	t-score = -1.13, df = 1, 113	.26
**Child's Age (in months)**	.33 (.02, .64)	t-score = 2.08, df = 1, 114	.04
**Number of Siblings**	.14 (-.28, .57)	t-score = .68, df = 1, 114	.50
**Number of Household Members**	.15 (-.05, .35)	t-score = 1.47, df = 1, 113	.14
**Hours of physical activity (in a day)**	.19 (.01, .37)	t-score = 2.14, df = 1, 114	.04
**Hours of sleep (in a day)**	-.01 (-.30, .29)	t-score = -.06, df = 1, 114	.96
